# Potential therapeutic applications of infusions and hydroalcoholic extracts of Romanian glutinous sage (*Salvia glutinosa* L.)

**DOI:** 10.3389/fphar.2022.975800

**Published:** 2022-08-19

**Authors:** Alexandru Nicolescu, Mihai Babotă, Maria Ilea, Maria Inês Dias, Ricardo C. Calhelha, Laura Gavrilaș, Gabriele Rocchetti, Gianina Crișan, Andrei Mocan, Lillian Barros, Alina Elena Pârvu

**Affiliations:** ^1^ Department of Pharmaceutical Botany, “Iuliu Hațieganu” University of Medicine and Pharmacy, Cluj-Napoca, Romania; ^2^ Department of Pathophysiology, Faculty of Medicine, “Iuliu Hațieganu” University of Medicine and Pharmacy, Cluj-Napoca, Romania; ^3^ Centro de Investigação de Montanha (CIMO), Instituto Politécnico de Bragança, Bragança, Portugal; ^4^ Department of Bromatology, Hygiene, Nutrition, “Iuliu Hațieganu” University of Medicine and Pharmacy, Cluj-Napoca, Romania; ^5^ Department of Animal Science, Food and Nutrition, Università Cattolica del Sacro Cuore, Piacenza, Italy; ^6^ Laboratory of Chromatography, Institute of Advanced Horticulture Research of Transylvania, University of Agricultural Sciences and Veterinary Medicine, Cluj-Napoca, Romania

**Keywords:** Salvia glutinosa, anti-inflammatory, ROS, antioxidant, alpha-glucosidase, polyphenols

## Abstract

**Ethnopharmacological relevance:** *Salvia glutinosa*, also known as the glutinous sage, has been used in Romanian folk medicine in the treatment of inflammation, injuries, and mild infections. However, there is no direct scientific evidence to demonstrate these activities.

**Aim of the Study:** The present research was based on evaluating antioxidant, antiproliferative, and α-glucosidase inhibitory activity of *S. glutinosa* extracts, as well as the *in vivo* anti-inflammatory activity.

**Materials and Methods:** Infusions and 70% (*v:v*) ethanol solution extracts of *S. glutinosa* stems and leaves, collected from two different locations in Romania, were prepared. Ten phenolic compounds were identified and quantified using the LC-DAD-ESI/MS^n^ method, and total phenolic and flavonoid content, as well as *in vitro* antioxidant (DPPH, ABTS, and FRAP assays), antiproliferative, anti-inflammatory and alpha-glucosidase inhibitory activities were determined. A rat model of induced inflammation with turpentine oil was used for the examination of *in vivo* effects of the extracts, using diclofenac as an anti-inflammatory control.

**Results:** The highest inhibitory α-glucosidase activity was determined to be IC_50_ = 0.546 mg/ml for the hydroalcoholic extract made with plant material collected on the road to Sighișoara. The highest cytotoxic activity against HepG2 cell line was determined to be GI_50_ = 131.68 ± 5.03 μg/ml, for the hydroalcoholic extract made with plant material from Sighișoara. *In vivo* administration of extract (200 mg lyophilized powder/ml) showed a significant reduction of NO production.

**Conclusion:** Our findings indicate that *S. glutinosa* extracts exhibit antioxidant, α-glucosidase inhibitory activity, as well as a modest cytotoxic effect on HepG2 cell line. By *in vivo* administration, the extracts show anti-inflammatory and antioxidant activity, which correlates with the traditional use of the species. The environmental conditions seemed to induce important changes in the chemical composition and the bioactivity of the herbal preparations derived from *S. glutinosa.*

## 1 Introduction

Dealing with a fascinating number of plant species with proven medicinal qualities, it seems that plants have been and continue to be extremely promising tools in the fight against a large diversity of diseases. In the Lamiaceae family, the genus *Salvia* became iconic for the variety of applications that it has provided throughout the centuries, including its high medicinal value and cosmetic and ornamental usage (Drew et al., 2017; Mocan et al., 2020). The dynamism of this genus comes from the fact that it comprises more than 1,000 species, and the number could be rising because of the fact that their taxonomical characteristics are still under close investigation, some genera even having been recently included in the genus (Drew et al., 2017).

The species of *Salvia* could provide many health benefits, but a great number of them are yet to be further investigated for their potential. A very prominent representant, *Salvia officinalis* L. has been extensively studied in terms of chemical composition, fragrance, large-scare production, culinary value and pharmacological properties (Cvetkovikj et al., 2013; Fu et al., 2013).

**FIGURE 1:**
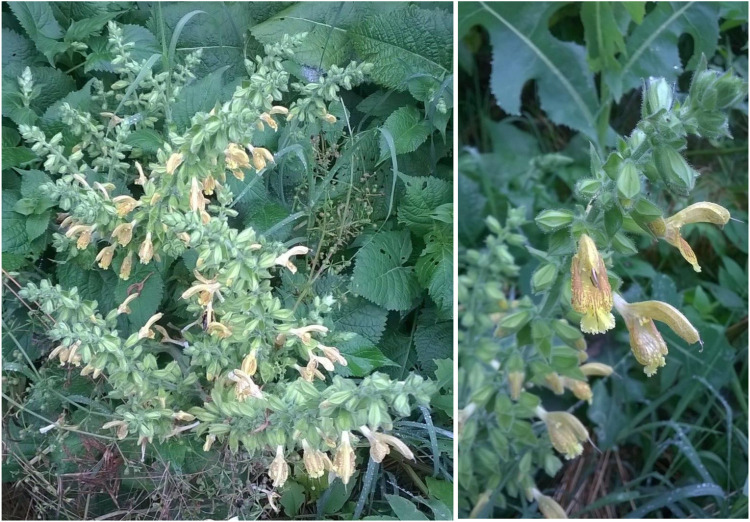
Appearance of *S. glutinosa* aerial parts, which have been used for extraction.

The vast range of sage species entails many yet unexplored species, and among them there is *Salvia glutinosa* L. (SG)—the glutinous sage, which is also known as Jupiter’s epitaph (Figure 1). There are some studies regarding the chemical composition and the structural aspects of this species, but there are still many factors that could be considered in order to build a much more detailed profile in relation to its therapeutic evaluation (Veličković et al., 2002; Velickovic et al., 2003; Mocan et al., 2020).

In Eastern Europe, specifically in Romania, there are several ethnopharmacological uses for SG. For example, in the *Encyclopedia of Romanian Ethnobotany*, Butură mentions the traditional usage of the blossomed plant, in both human and veterinary medicine (Butură, 1979). In human folk medicine, SG was used internally as root decoction (for dizziness) and as flower and leaf infusion (for gastroenteritis with abdominal pain, cough, and sweating). As topical treatment, SG was traditionally used as stem, leaf and flower decoction for headaches and rheumatism. The herbal remedy was also used for veterinary purposes, in the treatment of skin injuries, bites and feet pain in animals (Butură, 1979; Scarlat and Tohăneanu, 2019; Drăgulescu and Mărculescu, 2020). Moreover, SG herbal tea has been used traditionally in certain villages of Transylvania for throat inflammations (Papp et al., 2011). In other parts of the world (specifically, in India and Himalaya), the leaves of the plant are used to obtain a gargle that acts as a treatment for mouth ulcers and sore throat (Quattrocchi, 2012). In Italy, SG leaves are not only used as aromatic herb (Motti, 2021), but also as an alternative to *S. officinalis*, in the treatment of headache and angina pectoris (as digestive infusion) and for fevering sweats (as topical infusion). Moreover, abdominal pains can be healed using a decoction of leaves (Idolo et al., 2010).

There are some species of sage that have been previously studied for their potential for prevention and treatment of diabetes. *Salvia miltiorrhiza* Bunge (Danshen), one example of species with usage in the traditional Chinese medicine, has been observed to be beneficial due to its anti-diabetic effect, possibly mediated by the composition in salvianolic acids and diterpenoids (Jia et al., 2019; Orgah et al., 2020).

Thus, the aim of the present study was to evaluate the potential health benefit of the extracts of glutinous sage and to consider which would be the possible uses for this plant, in comparison to the sage species that are already considered to be extremely valuable sources for human consumption. The phenolic composition and the biological activity of aqueous and hydroethanolic extracts (obtained by infusion and maceration, respectively) made from SG collected from two different places in Romania (from Vâlcea and Brașov county) have been examined. The LC-DAD-ESI/MS^n^ technique has been used for the characterization of the qualitative and quantitative profile of certain phenolic compounds from the extracts, which was followed by *in vitro* analysis of antioxidant, antiproliferative and antidiabetic activities. Additionally, *in vivo* anti-inflammatory potential of the SG extracts was tested using a previously established rodent model.

## 2 Materials and methods

### 2.1 Chemical reagents and cell lines

Acetonitrile (99.9%) of HPLC grade was purchased from Fisher Scientific (Lisbon, Portugal). Phenolic compound standards (apigenin-6-*C*-glucoside, caffeic acid, quercetin-3-*O*-glucoside, and rosmarinic acid) were from Extrasynthèse (Genay, France). All the other general laboratory reagents were acquired from Panreac Química S. L. U. (Barcelona, Spain).

ABTS [2,20′azino-bis(3-ethylbenzothiazoline-6-sulfonic acid) diammonium salt] ≥ 98% purity, potassium peroxodisulfate (≥99% purity), DPPH (2,2-diphenyl-1-picrylhydrazyl), ferric chloride, TPTZ [2,4,6-Tris (2-pyridyl)-*s*-triazine], Trolox (6-hydroxy-2,5,7,8-tetramethyl-chromane-2-carboxylic acid; ≥97% purity), α-glucosidase, acarbose and fetal bovine serum (FBS) used in antioxidant and enzyme inhibition assays, were purchased from Sigma Aldrich Chemie GmbH (Steinheim, Germany). A Millipore Milli-Q Plus water treatment system was used to obtain Ultra-pure water (Millipore Bedford Corp., Bedford, MA, United States). All the other reagents used, including solvents, were of analytical grade (Mocan et al., 2020; Babotă et al., 2021).

The cell line MCF-7 (breast adenocarcinoma) was acquired from DSMZ (Leibniz-Institut DSMZ—Deutsche Sammlung von Mikroorganismen und Zellkulturen GmbH), and NCI-H460 (non-small cell lung cancer), HeLa (cervical carcinoma), and HepG2 from European collection of cell cultures (ECACC). For the antiproliferative assay, ellipticine and sulforhodamine B were purchased from Sigma Chemical Co. (Saint Louis, United States) (Calhelha et al., 2014).

For the *in vivo* determination of anti-inflammatory effect, the used reagents (thiobarbituric acid, vanadium (III) chloride, xylenol orange, [o-cresosulfonphtalein-3,3-bis (sodium methyliminodiacetate)], ortho-dianisidinedihydrochloride (3,3′- dimethoxybenzidine), ferrous ammonium sulfate, hydrogen peroxide, trichloroacetic acid (TCA)) were purchased from BioMaxima S. A., Lublin, Poland (Farcas et al., 2019).

### 2.2 Plant material

The aerial parts of SG samples were collected from Vâlcea county (45°06′56.9″N and 24°18′07.4″E; 280–300 m above sea level) and on the road to Sighișoara, in Brașov county (46°02′07.3″N 25°15′15.7″E; 450–470 m above sea level) in Romania, in June 2014 and in July 2018 respectively. The authenticity of plant material was validated after the identification procedure, which was accomplished by Dr. Andrei Mocan, from the Department of Pharmaceutical Botany, Faculty of Pharmacy, “Iuliu Hațieganu” University of Medicine and Pharmacy in Cluj-Napoca, Romania, where voucher specimens have been deposited (voucher numbers 436 and 490 respectively). The selection of plant material was performed to obtain representative samples without damaging the natural population. Samples were dried at room temperature and then properly stored until the extraction, at constant temperatures (20 ± 2°C). The plant samples were ground to a fine powder, at 6,000 rpm and for 1.5 min, using a laboratory mill and then subjected directly to the extraction procedure.

### 2.3 Extraction procedure

For this study, we have taken into consideration two types of extracts: ethanolic and water extracts, which were accomplished by *infusion* (water extraction at heat) and *maceration* (hydroalcoholic extraction at room temperature and in the dark).

*Maceration*: 1 g of previously weighed SG powder was added over 100 ml of 70% (*v:v*) ethanol solution, in an Erlenmeyer flask, being shaken and kept at room temperature in a place without light for 10 days. The hydroethanolic extracts were filtered, cooled at room temperature, and subjected to alcohol evaporation under reduced pressure, using a rotary evaporator. The remaining extract was subsequently lyophilized to remove the remaining water, obtaining a dry SG hydroalcoholic extract (SGE). To distinguish the two types of probes, SGE were divided into SG1 and SG2, which account for the extracts obtained from plant material originating in Vâlcea and Sighișoara, respectively.

*Infusion*: 10 g of previously weighed SG powder was added over 1 L of boiling water, the mixture being continuously stirred for 30 min. The aqueous extracts were filtered, cooled at room temperature, and subsequently lyophilized to remove the remaining water, obtaining a dry SG infusion (SGI). To distinguish the two types of probes, SGI were divided into SG3 and SG4, representing the extracts obtained from plant material originating in Vâlcea and Sighișoara, respectively.

Final solid lyophilizates were subsequently stored in a desiccator, at constant temperatures (20 ± 2°C) and in a dark place, until further analyses. For the phytochemical tests, TPC, TFC and antioxidant assays, the dry extracts were dissolved in the same solvent of extraction, 70% (*v:v*) ethanol solution and water respectively, excepting enzyme inhibition assays, where the freeze-dried hydroalcoholic extracts were resolubilized in 10% DMSO solution.

### 2.4 LC-DAD-ESI/mass spectrometer analysis of phenolic compounds

The phenolic profile was determined using the LC-DAD-ESI/MS^n^ method (Dionex Ultimate 3000 UPLC, Thermo Scientific, San Jose, CA, United States). The separation and identification of several compounds was accomplished using a previously described method (Bessada et al., 2016). The obtained extracts were re-dissolved at a concentration of 10 mg/ml with water and with an ethanol: water (70:30, *v/v*) mixture for the infusions and for the hydroalcoholic extracts respectively. A double online detection was performed using a DAD (280, 330, and 370 nm as preferred wavelengths) and a mass spectrometer (MS). The MS detection was performed in negative mode, using a Linear Ion Trap LTQ XL mass spectrometer (Thermo Finnigan, San Jose, CA, United States) equipped with an ESI source.

The identification of the phenolic compounds was performed based on their chromatographic behavior and UV-vis and mass spectra by comparison with standard compounds and with data reported in the literature, leading to a tentative identification. Data acquisition was achieved using an Xcalibur^®^ data system (Thermo Finnigan, San Jose, CA, United States). For quantitative analysis, a calibration curve for each available phenolic standard was constructed based on the UV-vis signal. In the case of identified phenolic compounds for which commercial standards were not available, the quantitative analysis was performed through the calibration curve of the most similar available standard. The results were expressed as mg/g of extract.

### 2.5 Total phenolic content and total flavonoid content

For the determination of TPC, the protocol was based on the Folin-Ciocalteu method, adapted to the microplate reader, which was previously reported by Babotă et al. (2018). After 30 min of incubation at room temperature, the absorbance of the samples was read at 760 nm. The results were expressed as milligrams of gallic acid equivalents per gram of freeze-dried powder (mg GAE/g dw). For the determination of TFC, the aluminium chloride method was used (Babotă et al., 2018), results being expressed as milligrams of rutin equivalents per gram of freeze-dried powder (mg RE/g dw). For both of the assays, samples were re-dissolved in the original solvent, and then further diluted and analyzed in 96-well plates using a SPECTROstar^®^ Nano Multi-Detection Microplate Reader (BMG Labtech, Ortenberg, Germany).

### 2.6 Determination of antioxidant capacity

The antioxidant potential of SGI and SGE was tested through two complementary methods (DPPH and ABTS or TEAC, indicating the radical scavenger activity) and through FRAP (ferric reducing antioxidant power), the protocols being previously described by Mocan et al. (2017), Babotă et al. (2018), and Rusu et al. (2018). Samples were re-dissolved in 70% ethanol solution or distilled water, obtaining a concentration of 1 mg/ml, which was further diluted; the obtained probes were further analyzed in 96-well plates using a SPECTROstar^®^ Nano Multi-Detection Microplate Reader (BMG Labtech, Ortenberg, Germany).

*DPPH (1,1-diphenyl-2-picrylhydrazyl) radical scavenging assay:* 30 μl of each sample were mixed with a 0.004% methanol solution of DPPH. After 30 min of incubation at room temperature in the dark, the absorbance was read at 517 nm. The results of DPPH radical scavenging activity were expressed as mg of Trolox equivalents per gram of freeze-dried powder (mg TE/g dw) (Babotă et al., 2018).

*ABTS* [*2,2′-azino-bis(3-ethylbenzothiazoline) 6-sulfonic acid*] *radical scavenging assay:* ABTS^+^ was obtained directly by reacting 7 mM ABTS solution with 2.45 mM potassium persulfate and letting the mixture stand for 12–16 h in the dark at room temperature. Before the assay, ABTS solution was diluted with distilled water until reaching an absorbance of 0.700 ± 0.02 at 734 nm. Diluted samples were mixed with ABTS solution and mixed. After 30 min of incubation at room temperature, the sample absorbances were read at 734 nm. The results of ABTS radical scavenging activity were expressed as mg of Trolox equivalents per gram of freeze-dried powder (mg TE/g dw) (Babotă et al., 2018).

*FRAP* (Ferric reducing antioxidant power) was tested using the FRAP reagent, obtained by mixing acetate buffer (0.3 M, pH 3.6), 2,4,6-tris(2-pyridyl)-*s*-triazine (TPTZ) (10 mM) in 40 mM HCl and ferric chloride (20 mM) in a ratio of 10:1:1 (*v/v/v*). The absorbance was read at 593 nm after a 30 min incubation at room temperature, and the results were expressed as mg of Trolox equivalents per Gram of freeze-dried powder (mg TE/g dw) (Babotă et al., 2018).

### 2.7 *In vitro* α-glucosidase inhibition assay

The antidiabetic properties of the extracts were determined based on their *in vitro* inhibitory capacity on α-glucosidase, using a previously described method (Tanase et al., 2019). Briefly, 50 μl of diluted samples and 50 μl of 100 mM-phosphate buffer (pH 6.8) were mixed with 50 μl of yeast α-glucosidase in a 96-well microplate for 10 min, and then 50 μl of substrate (5 mM, p-nitrophenyl-α-D-glucopyranoside, prepared in the same buffer) was added. The coloration due to the formation of p-nitrophenol was measured at 405 nm, after incubation at 37°C. The blanks for test samples were also prepared, using a positive control with the highest absorbance, and a series of concentrations of acarbose were used as an inhibition standard. The percentages of inhibition (I) for a serial dilution of samples were calculated using the formula:

$$I\;\left(\%\right)=100*\frac{Abs_{control}-Abs_{sample}}{Abs_{control}}$$

The results were expressed as IC_50_ (the concentration of the sample that was able to inhibit 50% of the enzyme), using the normalized logarithmic curve for the determined percentages of inhibition, and the dependence between the logC and the I (%) was graphed using Prism 8 (GraphPad).

### 2.8 *In vitro* antiproliferative activity

Firstly, the extracts were re-dissolved in water at a 8 mg/ml concentration and further diluted in the range of 400 to 6.25 μg/ml. The cytotoxic properties were evaluated using four human tumor cell lines: MCF-7 (breast adenocarcinoma), NCI-H460 (non-small cell lung cancer), HeLa (cervical carcinoma), and HepG2 (hepatocellular carcinoma). Consequently, a non-tumor cell line (PLP2) was evaluated using a procedure previously described by Abreu et al. (2011). The Sulforhodamine B assay was accomplished using the method described by Barros et al. (2013), with Ellipticine as positive control, and also a negative control was provided by each suspension of cells. The results were expressed in GI_50_ values (the concentration that was able to inhibit the proliferation of 50% of the cells).

### 2.9 Anti-inflammatory activity

The extracts were re-dissolved in water at a 8 mg/ml concentration and then diluted in the range of 400 to 6.25 μg/ml. In this study, a mouse macrophage-like cell line RAW 264.7 was used, and the determination of nitric oxide was accomplished using the Griess Reagent System (GRS) kit, with measurements at 515 nm (ELx800 microplate reader, Bio-Tek Instruments, Inc.; Winooski, VT, United States), as previously described (Souilem et al., 2017). The results were expressed in IC_50_ values (the concentration of the sample that was able to provide the inhibition of 50% of NO production) and Dexamethasone was used as a positive control. For the negative controls, no LPS was added.

### 2.10 *In vivo* anti-inflammatory activity

#### 2.10.1 Experimental animals

For the *in vivo* study was used the plant product with the best *in vitro* antioxidant activity and phytochemical characteristics, which was the hydroalcoholic extract obtained with plant material from Sighișoara. The animals used for the experiments were adult male Wister albino rats (strain Crl: WI), weighing 200–250 g, and they were bred in the “Iuliu Hațieganu” University of Medicine and Pharmacy Animal Facility. During the study the animals were kept in a room under controlled temperature (22 ± 2°C) and humidity (50% ± 5%), subjected to a 12/12 h dark/light cycle, and with free access to food and water. At the end of the experiment, animals were killed by cervical dislocation under general anesthesia induced with ketamine (70 mg/kg b. w.) and xylazine (10 mg/kg b. w.) (Francischi et al., 2017). The experiments were performed in triplicate. Experimental design was approved by the Institutional Animal Ethical Committee (IAEC) of the “Iuliu Hațieganu” University of Medicine and Pharmacy Cluj-Napoca and by the National Sanitary Veterinary and Food Safety Agency (nr. 212/27.06.2019).

#### 2.10.2 Experimental protocol

The animals were randomly divided into nine groups (*n* = 5), as follows: negative control (CONTROL); inflammation (INFLAM); inflammation and diclofenac (10 mg/kg b. w.) treatment (DIC); inflammation and *S. glutinosa* extract (200 mg d. w./ml) treatment administrated in three dilutions (100%, 50%, 25%); *S. glutinosa* extract (200 mg d. w./ml) treatment administrated in three dilutions (100%, 50%, 25%). Inflammation was induced by intramuscular (i. m.) injection with turpentine oil (0.6 ml/kg b. w.) in day one (Toyohara et al., 2013). The treatments were administrated orally by gavage (1 ml/day) for 7 days starting with day one. Animals from the CONTROL and INFLAM groups received by gavage tap water (1 ml/animal/day) for 7 days (Andreicut et al., 2018). In day 8, blood samples were collected by retro-orbital puncture, carried out under general anesthesia, and then serum was separated and stored at −80°C until use, and animals were euthanized by cervical dislocation.

#### 2.10.3 Oxidative stress parameters evaluation

Oxidative stress was firstly assessed using global oxidative stress assays, respectively total oxidative status (TOS), total antioxidant reactivity (TAR), and the oxidative stress index (OSI). TOS was assessed using a colorimetric method, and results were expressed in μmol of H_2_O_2_ equiv./L (Erel, 2005). TAC has also been measured using a colorimetric assay, and results were expressed as mmol Trolox equiv./L (Erel, 2004). OSI was calculated as the ratio between TOS and TAC (Harma et al., 2003).

Specific oxidative stress tests, such as malondialdehyde (MDA), total thiols (SH), and total serum nitrates and nitrates (NOx), were also performed (Farcas et al., 2019). MDA was determined by using the thiobarbituric acid assay, and results were expressed as nmol/mL (Draper et al., 1993). The serum total nitrites and nitrates concentration was assessed using the Griess reaction, and results were expressed as nitrite μmol/L (NOx) (Miranda et al., 2001; Ghasemi et al., 2007). Total serum thiols (SH) were determined using Ellman’s reagent, and results were expressed as mmol GSH/mL (Hu, 1994).

## 3 Results and discussions

### 3.1 LC-DAD-ESI/mass spectrometer quantitative and qualitative analysis

The chromatographic screening of *S. glutinosa* extracts allowed us to confirm the presence of ten phenolic compounds belonging to four major classes: *phenolic acids*–glycosides of monomers (compound **1**), dimers (compound **6**) and trimers (compounds **9** and **10**), *flavones* (compounds **2**, **7**, and **8**), *flavonols* (compounds **3** and **5**) and *lignans* (compound **4**) (Table 1). Compound **1** ([M−H]^−^ at *m/z* 341) yielded a base peak at *m/z* 179 (corresponding to deprotonated caffeic acid), being assigned as caffeic acid hexoside (after losing an hexosyl moiety, 162 u). In a similar way, compounds **3** ([M−H]^−^ at *m/z* 463) and **5** ([M−H]^−^ at *m/z* 477) formed MS^2^ molecular ions with *m/z* 301—[quercetin−H]^−^ and *m/z* 315—[isorhamnetin−H]^−^, losing hexosyl moieties and based on the spectral and mass characteristics of these fragments, they were assigned as quercetin and isorhamnetin *O*-hexoside, respectively. Compound **2** showed a fragmentation pattern specific for *C*-flavonoids previously described (Becchi and Fraisse, 1989), releasing two fragments with *m/z* 120 (corresponding to consecutive cleavage of hexosyl moieties linked to six and eight positions of apigenin aglycone), the structure being tentatively identified as apigenin-*C*-dihexoside. Rosmarinic acid **6**) ([M−H]^−^ at *m/z* 359) exerted a λ_max_ at 331 nm, its identity being further confirmed by comparison with an authentic standard, while baicalein **7**) ([M−H]^−^ at *m/z* 269) was also identified based on main fragment ions resulted after MS^2^ fragmentation. Compound **4** showed a molecular ion [M−H]^−^ at *m/z* 463 and λ_max_ at 341 nm, releasing common fragment ions (*m/z* 359, m*/z* 197, m*/z* 179, and m*/z* 161) with rosmarinic acid (**6**), being tentatively identified as sagerinic acid; both compounds were cited as main constituents of *Salvia* species, with the mention that sagerinic acid exerted a fragmentation pattern specific for cyclobutane-type lignans (bioactive secondary metabolites common for Lamiaceae species) (Yu et al., 2017; Sharma et al., 2020). Compounds **9** and **10** produced, after MS^2^ fragmentation, the common abundant ion fragment with *m/z* 295, which indicated the loss of a deprotonated 3-(3,4-dihydroxyphenyl) lactic acid unit (known as danshensu), describing a characteristic fragmentation for salvianolic acids with trimeric structure (Liu et al., 2007). Hence, corroborated with the others chromatographic features of each compound, peak **9** was tentatively assigned as salvianolic acid J, while peak **10** as salvianolic acid A.

**TABLE 1:** Chromatographic features, qualitative and quantitative distribution (mg/g of extract) of the phenolic compounds present in extracts from *Salvia glutinosa* L.

Peak	Rt (min)	λ_max (nm)_	Molecular ion [M-H]^-^ (*m/z*)	MS^2^ (*m/z*)	Tentative identification	Quantification			
						*SG1*	*SG2*	*SG3*	*SG4*
1	6.56	323	341	179 (100), 135 (32)	Caffeic acid hexoside	0.44 ± 0.01	tr	1.47 ± 0.03	0.71 ± 0.00
2	8.72	343	593	473 (100), 383 (38), 353 (81)	Apigenin-*C*-dihexoside	0.81 ± 0.02	0.95 ± 0.00	1.13 ± 0.02	0.51 ± 0.04
3	14.96	328	463	301 (100)	Quercetin-*O*-hexoside	0.65 ± 0.01	0.58 ± 0.01	0.68 ± 0.03	0.88 ± 0.01
4	17.84	341	719	539 (21), 521 (12), 359 (89), 197 (18), 179 (30), 161 (82)	Sagerinic acid	2.39 ± 0.07	2.72 ± 0.07	4.01 ± 0.02	5.09 ± 0.05
5	18.94	342	477	315 (100)	Isorhamnetin-*O*-hexoside	1.86 ± 0.03	3.26 ± 0.02	1.48 ± 0.00	2.60 ± 0.09
6	20.31	331	359	359 (37), 197 (63), 179 (81), 161 (100)	Rosmarinic acid	40.63 ± 0.81	39.71 ± 0.20	31.05 ± 0.35	83.77 ± 0.19
7	21.67	334	269	251 (100), 241 (32), 223 (12), 197 (5)	Baicalein	1.30 ± 0.05	1.16 ± 0.02	1.19 ± 0.06	1.90 ± 0.15
8	22.72	331	489	447 (5), 285 (42)	Luteolin acetyl-glucoside	5.00 ± 0.06	3.99 ± 0.07	2.94 ± 0.03	3.26 ± 0.06
9	25.35	321	537	49 3 (100), 295 (83)	Salvianolic acid J	2.29 ± 0.06	6.28 ± 0.22	4.24 ± 0.04	3.55 ± 0.03
10	25.85	322	493	313 (34), 295 (100), 203 (5)	Salvianolic acid A	2.86 ± 0.19	3.27 ± 0.06	2.65 ± 0.04	3.36 ± 0.04
Total phenolic compounds						58.23 ± 0.13	61,93 ± 0.07	50,83 ± 0.06	105,63 ± 0.07

Tr–trace amounts. Standard calibration curves used for quantification: A–caffeic acid (*y* = 3,883,45*x* + 406,369, *R* ^*2*^ = 0.9939, LOD, 0.78 μg/ml; LOQ, 1.97 μg/ml); B–apigenin-6-*C*-glucoside (*y* = 1,973,37*x* + 30,036, *R* ^*2*^ = 0.9997, LOD, 0.19 μg/ml; LOQ, 0.63 μg/ml); C–quercetin-3-*O*-glucoside (y = 348,43x–160,173, *R* ^2^ = 0.9998, LOD, 0.21 μg/ml; LOQ, 0.71 μg/ml); and D–Rosmarinic acid (y = 1,912,91x–652,903, *R* ^2^ = 0.999, LOD, 0.15 μg/ml; LOQ, 0.68 μg/ml); . Values are expressed as mean ± SD (*n* = 3).

Regarding the quantitative distribution of the above-mentioned secondary metabolites identified in *S. glutinosa* herbal preparations (Table 1), it was observed that phenolic acids derivatives were the most abundant compounds quantified in the analyzed samples. Rosmarinic acid reached the highest concentration in SG4 (the infusion obtained from *S. glutinosa* harvested from Sighișoara), while SG2 (the hydroethanolic extract obtained from *S. glutinosa* harvested from Sighișoara) showed the most important content of salvianolic acid J; in a similar way, salvianolic acid A was quantified in high amounts in the extracts of the samples collected from the road to Sighișoara. The presence of salvianolic acids in *Salvia* species was previously correlated with their various bioactive properties (Liu et al., 2007; Ho and Hong, 2011); to the best of our knowledge, the present study highlights for the first time the presence of salvianolic acids A and J in *S. glutinosa* aerial parts. Moreover, luteolin acetyl-glucoside was quantified as the most abundant compound from all flavonoid derivatives found in *S. glutinosa*, its higher concentration being found in SG1 extract. It can be also observed that, from quantitative perspective, the samples collected from Sighișoara were richer in phenolic constituents than those collected from Vâlcea, revealing that environmental conditions can induce important changes in chemical composition of *S. glutinosa*. These findings encourage us to undergo further studies that can deeply explain the interdependence between growing conditions and variations in the phytochemical profile of these species.

### 3.2 Total phenolic content and total flavonoid content

Among all the bioactive compounds in the *Salvia* species, phenolic compounds, especially the flavonoids, are the most important (Mocan et al., 2020), Lamiaceae family being known for the rich composition in polyphenolic compounds, which may account for the significant antioxidant activity (Tosun et al., 2009). In the light of the importance of phenolic compounds, we aimed to evaluate the total phenolic and flavonoidic content of the SG extracts, and the obtained results are presented in Table 2.

**TABLE 2:** Comparison of total phenolic and flavonoid content, and *in vitro* antioxidant capacity (using the DPPH, ABTS, and FRAP assays) of the extracts of *S. glutinosa*.

Sample	Extraction solvent	TPC (mg GAE/g dw)	TFC (mg RE/g dw)	DPPH scavenging (mg TE/g dw)	ABTS scavenging (mg TE/g dw)	FRAP (mg TE/g dw)
SG1	SGE	169.14 ± 6.80	22.75 ± 1.20	122.70 ± 2.86	177.24 ± 6.51	624.76 ± 10.91
SG2	70% ethanol (*v/v*)	178.65 ± 2.97	30.16 ± 0.62	123.49 ± 4.25	163.88 ± 11.63	737.41 ± 34.46
SG3	SGI	206.87 ± 5.20	18.21 ± 1.07	65.29 ± 5.77	397.92 ± 12.45	575.95 ± 12.95
SG4	Water	228.31 ± 5.32	24.24 ± 0.64	66.02 ± 5.18	422.32 ± 13.09	621.00 ± 10.95

Note: results are presented as mean ± SD (standard deviation) of three parallel measurements.

**FIGURE 2:**
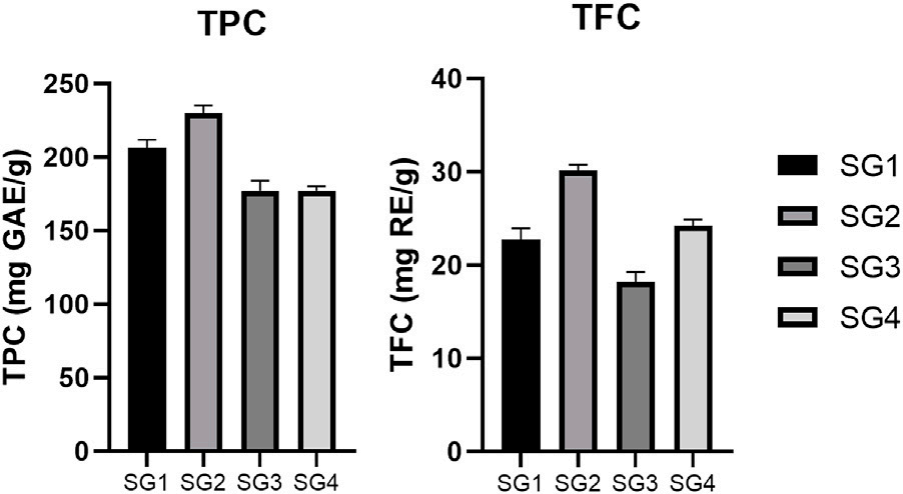
Comparison between total phenolic content (TPC) and total flavonoid content (TFC) determined for the SG samples. The results are expressed as relative means ± standard deviation of three parallel measurements.

The highest TPC was determined for the powder originating in the infusions (228.31 ± 5.32 mg GAE/g dw for the plant from Sighișoara, respectively 206.87 ± 5.20 mg GAE/g dw for the plant from Vâlcea). The lowest concentrations were observed for the hydroalcoholic freeze-dried powder (178.65 ± 2.97 mg GAE/g dw for the plant from Sighișoara, respectively 169.14 ± 6.80 mg GAE/g dw for the plant from Vâlcea) (Figure 2). The slight difference in the results shows that same species growing in two different geographical locations exhibit a difference in phytochemical composition. Regarding the total phenolics, the samples collected from Sighișoara showed a higher concentration, for every type of extract. In a previous study, Veličković et al. analyzed the TPC of SG 70% ethanol (v/v) extracts, and the results varied from 121.0 ± 0.49 mg gallic acid/g of dry extract to 137.3 ± 0.42 mg gallic acid/g of dry extract, depending on the extraction method (Veličković et al., 2011), similar to our results. Regarding the extractability of phenolic compounds, the results of our study are supported by Dent et al., which found that a 70% ethanol/water solvent mix is less efficient than just water in the case of *S. officinalis*, according to the relative polarity of the compounds (Dent et al., 2013). Moreover, the situation was similar for other plant species, and water extracts showed higher TPC levels than ethanolic extracts of *Nephelium mutabile* (Sopee et al., 2019) and *Punica granatum* (Zam et al., 2012), due to the polarity of the phenolic compounds. The water extracts have been realized at temperature, which is another factor that could have contributed to the higher yield of phenolics in SGI. This finding is supported by Torun et al. (2015) which have observed that the TPC significantly increased with temperature in the case of *Salvia fruticosa* extracts, probably due to the cell wall disruption or by the rise in extractability as a result of transforming insoluble compounds into soluble ones (both being heat-mediated processes) (Torun et al., 2015).

On the other hand, the highest TFC was determined for the powder originating in the hydroalcoholic extracts (30.16 ± 0.62 mg RE/g dw for the plant from Sighișoara, respectively 22.75 ± 1.20 mg RE/g dw for the plant from Vâlcea). The lowest concentrations were observed for the aqueous freeze-dried powder (24.24 ± 0.64 mg RE/g dw for the plant from Sighișoara, respectively 18.21 ± 1.07 mg RE/g dw for the plant from Vâlcea) (Figure 2). As in the case of TPC, the obtained TFC values were slightly different for SG originating in different geographical locations, once again highlighting the importance of the environmental conditions. Veličković et al. (2007) showed that for SG, there is a higher yield of flavonoids when polar solvents are used in comparison to non-polar ones (70% ethanol and respectively petroleum ether), and that 70% ethanol induced a higher TFC compared to using water as solvent. Hence, our results are in line with previous studies on SG, showing that existing flavonoids (for example, flavones such as apigenin and luteolin) are less extractible in water than in a slightly less polar solvent.

Considering the aforementioned factors, a combination of parameters could be used in the future to maximize extractability of the total phenolics and flavonoids in SG. For example, in the case of *Salvia officinalis*, Duletić-Laušević et al. (2019) have observed that the highest TPC and TFC values were obtained for extracts realized with 50% ethanol solution, in comparison to using only water or 96% ethanol. In conclusion, there is a slight difference between SG samples collected from two different geographical locations regarding total phenolics and flavonoids. The environmental conditions can be one of the factors responsible for the composition in bioactive compounds, as some authors have previously noted (Adhikari et al., 2018).

### 3.3 Antioxidant activity

The *in vitro* antioxidant potential of the obtained SG extracts was evaluated using three complementary assays, as presented in Table 2.

The obtained results show variation for each assay, however, excepting ABTS in the case of SG2 sample, the antioxidant capacity is correlated with the total content of phenolic and flavonoid compounds. This could be linked to the fact that phenolic compounds in sage species (and generally for the representants of Lamiaceae family) have been shown to exhibit significant scavenging activity on oxygen reactive species (Santos-Gomes et al., 2002; Tosun et al., 2009). Comparing nine sage species, Loizzo et al. (2014) concluded that SG methanolic extracts showed the highest antioxidant activity for DPPH (IC_50_ of 3.2 ± 0.3 μg/ml), ABTS (59.1 ± 0.4 μmol TE/g of dried extract) and FRAP (422.0 ± 9.8 μmol Fe^2+^ equivalents/g of dried extract) assays, followed by *S. sclarea, S. hydrangea* and *S. ceratophylla,* which was also correlated with the values for TPC and TFC. Regarding the SG extracts, the best antioxidant activity was proven for the hydroalcoholic extracts, except for ABTS assay, which showed higher values for infusions.

**FIGURE 3:**
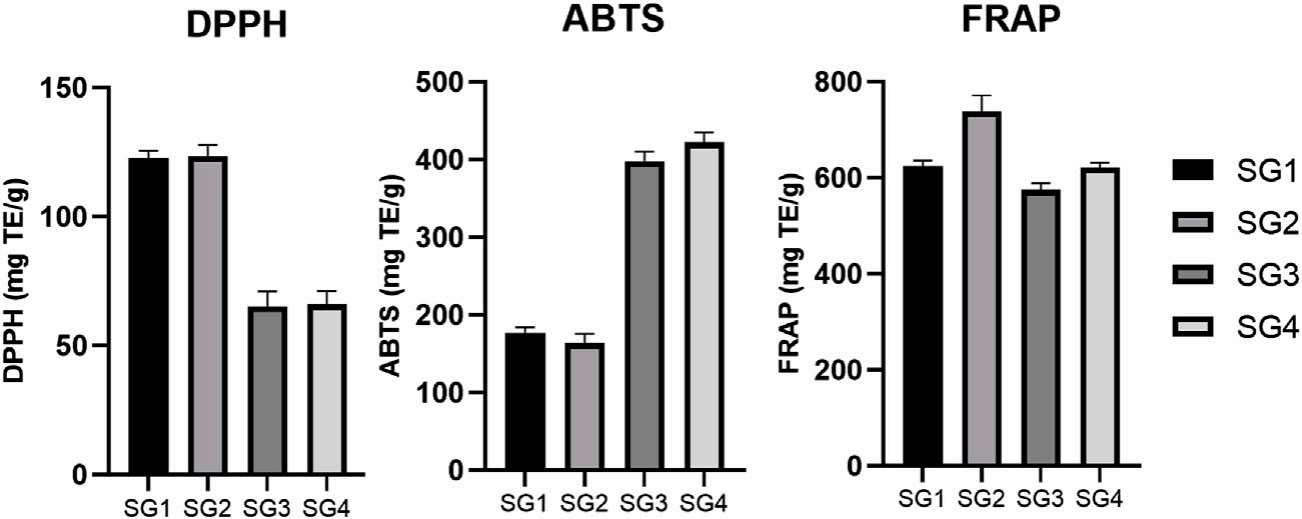
Comparison between the DPPH, ABTS, and FRAP *in vitro* antioxidant activity determined for the SG samples. The results are expressed as relative means ± standard deviation of three parallel measurements.

In the case of DPPH (Figure 3), there was a higher activity determined for SGE (123.49 ± 4.25 mg TE/g of freeze-dried powder for SG2 and 122.70 ± 2.86 mg TE/g of freeze-dried powder for SG1) and a lower activity for SGI (66.02 ± 5.18 mg TE/g of freeze-dried powder for SG4 and 65.29 ± 5.77 mg TE/g of freeze-dried powder for SG3). There is a negligible difference in activity between the plants from different geographical locations and the same solvent used for extraction, but the results show that plant material from Sighișoara showed a slightly higher activity. For ABTS (Figure 3), the highest activity was recorded for the aqueous SG4 and SG3 (422.32 ± 13.09 mg TE/g of freeze-dried powder for SG4 and 397.92 ± 12.45 mg TE/g of freeze-dried powder for SG3). Interestingly, determinations by ABTS assay show higher activity for SGI that for SGE, for which the highest activity was 177.24 ± 6.51 mg TE/g of freeze-dried powder.

Moreover, the greater values for ABTS than for DPPH seem to be supported by a previous study, where 70% ethanolic extraction of SG aerial parts showed an activity of 80.42 ± 0.95 mg TE/g extract for DPPH and 126.70 ± 5.22 mg TE/g extract for ABTS (Mocan et al., 2020). Even though the results of ABTS and DPPS assays seem to be in opposition, the explanation might arise from a theory that was previously described. Due to the high complexity of phytochemical matrices, the qualitative and quantitative analysis of the chemical compounds present in plants is suspected to high variability (Carocho and Ferreira, 2013). Thus, *in vitro* antioxidant assays could be highly influenced by these variations (Carocho and Ferreira, 2013). Nevertheless, it seems that, at least for SG, a higher polarity of solvent (for example, when comparing methanol with ethyl acetate) is correlated with a higher DPPH and ABTS scavenging activity (Miliauskas et al., 2004).

Ferric reducing antioxidant power (Figure 3) was the highest for the extracts obtained from plants originating in Sighișoara and higher for the hydroalcoholic samples (737.41 ± 34.46 mg TE/g of freeze-dried powder for SG2 and 624.76 ± 10.91 mg TE/g of freeze-dried powder for SG1) and the lowest was for SG3 (575.95 ± 12.95 mg TE/g of freeze-dried powder).

### 3.4 α-glucosidase inhibition activity

Luteolin and its derivatives have been shown to have an inhibitory activity on α-glucosidase (Kim et al., 2000). Since luteolin acetyl-glucoside has been successfully identified in this study using the LC-DAD-ESI/MS^n^ method, we further tested the α-glucosidase inhibition activity. Among the enzymes used in the gastro-intestinal tract, glycosidases are one of the most important, being involved in many anabolic and catabolic processes. Considering the particular case of α-glucosidase, compounds that are able to inhibit this enzyme have a broad-spectrum therapeutic potential, especially in the treatment of diabetes mellitus (Gowri et al., 2007). The extracts were evaluated for their potential benefit as antidiabetic agents.

**FIGURE 4:**
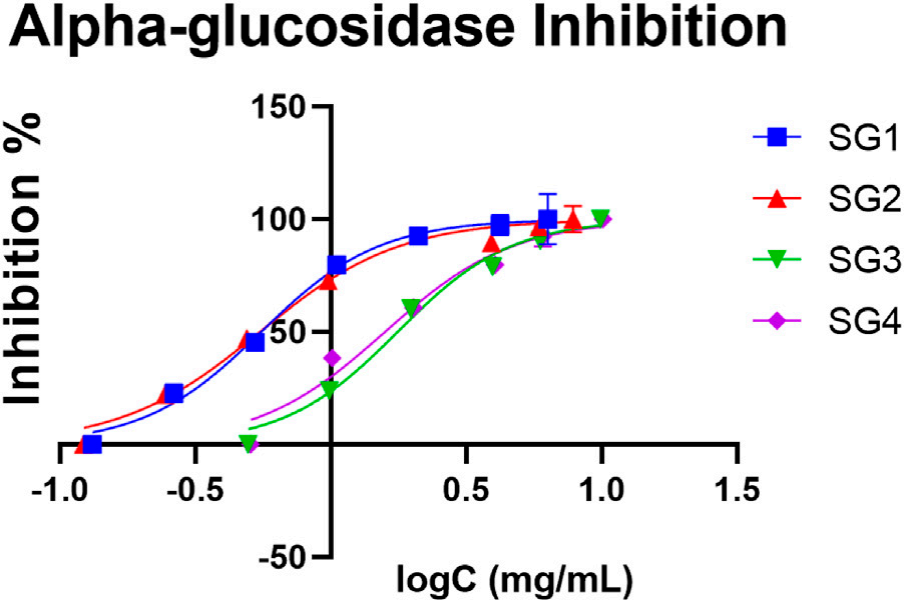
The concentration (expressed as logC, in mg/mL) dependent inhibition effect of *S. glutinosa* extracts on yeast α-glucosidase. The IC_50_ for acarbose was 0.239 mg/ml. Each point indicates average ±SD (standard deviation) of three parallel measurements.

In comparison to SGI, SGE displayed a higher α-glucosidase inhibitory capacity. Interestingly, the IC_50_ values for SG1 and SG3 were slightly higher in comparison to SG2 and SG4, which suggest there might be a difference in activity for the plant material collected from Vâlcea and Sighișoara. The highest inhibitory activity was determined to be 0.546 mg/ml for the hydroalcoholic extract made with plant material from Sighișoara, and the value for the aqueous extract was 1.581 mg/ml. In the case of Vâlcea extracts, the inhibitory activity was determined to be 0.552 mg/ml for SG1 and 1.761 mg/ml for SG3. The determined IC_50_ for the acarbose standard was 0.239 mg/ml. The dependence of inhibition percent and the logarithm of concentration for every sample have been plotted in the graph in Figure 4, which clearly shows that hydroalcoholic extraction induced a higher activity, with an IC_50_ at lower concentrations. The inhibitory activity of the ethanolic extracts has been previously observed by Mocan et al., which found a IC_50_ of 21.54 ± 1.29 mmol acarbose equivalents/g of extract (Mocan et al., 2020).

Moreover, the quantitative analysis showed that there is a higher content of luteolin derivative in SGE (5.00 ± 0.06 mg/g for SG1 and 3.99 ± 0.07 mg/g for SG2) compared to SGI (2.94 ± 0.03 mg/g for SG3 and 3.26 ± 0.06 mg/g for SG4), which strengthens this hypothesis that there could be a link between the presence of this compound and the determined inhibitory activity, as observed in Table 1. Our study shows that the powder obtained from hydroalcoholic extracts of SG display a slightly lower activity than acarbose, but it could have the potential to be used as an alternative prevention method for diabetes, possibly due to the composition in salvianolic acids and other polyphenols.

### 3.5 *In vitro* antiproliferative and anti-inflammatory activity

There is a strong link between cancer and inflammation. Malignant cells trigger inflammatory and immune responses, and inflammatory non-malignant cells have tumour suppression activity. (Santos et al., 2018). These mechanisms impose the need to evaluate antiproliferative and anti-inflammatory activities together.

The results for the antiproliferative activity that were accomplished for four different human tumor cell lines (HeLa, HepG2, MCF-7, and NCI-H460, respectively) are shown in Table 3.

**TABLE 3:** Cytotoxic and anti-inflammatory activities of *S. glutinosa* extracts.

Sample	*SG1*	*SG2*	*SG3*	*SG4*
Cytotoxic activity1 (GI50; μg/ml)				
NCI H460 (non-small cell lung cancer)	263.96 ± 14.97	224.37 ± 16.91	229.44 ± 14.89	244.08 ± 17.23
MCF-7 (breast carcinoma)	180.85 ± 8.78	191.06 ± 8.12	234.52 ± 9.28	211.54 ± 8.89
HepG2 (hepatocellular carcinoma)	152.04 ± 8.85	131.68 ± 5.03	208.05 ± 9.23	177.38 ± 4.23
HeLa (cervical carcinoma)	205.67 ± 10.15	163.45 ± 7.22	293.79 ± 13.30	278.11 ± 11.40
PLP2 (porcine liver primary culture)	>400	>400	>400	>400
Anti-inflammatory activity2 (IC50; μg/ml)				
NOS production	304.93 ± 11.19	263.07 ± 11.62	>400	>400

Notes: ^1^GI_50_ values for ellipticine: 1.0 ± 0.09 μg/ml (NCI-H460), 0.91 ± 0.04 μg/ml (MCF-7), 1.1 ± 0.2 μg/ml (HepG2), 1.91 ± 0.06 μg/ml (HeLa), and 3.2 ± 0.7 μg/ml (PLP2); ^2^IC_50_ values for dexamethasone: 16 ± 1 μg/ml (NOS). Different letters in the same row mean significant differences (*p* < 0.05). Different letters in the same row mean significant differences (*p* < 0.05). *Means statistical differences obtained by a student *t*-test. Results are presented as mean ± SD (standard deviation) of three parallel measurements.

SGE displayed higher antiproliferative activity in comparison to SGI, which is characterized by a smaller value for GI_50_. The highest inhibition was determined for the HepG2 cell line, with 152.04 ± 8.85 μg/ml and 131.68 ± 5.03 μg/ml for SGE and 208.05 ± 9.23 μg/ml and 177.38 ± 4.23 μg/ml for SGI, respectively. Also, for SGE it is worth mentioning that the antiproliferative activity was notable for HeLa and MCF-7 cell lines. The activity of SGI on all the cell lines and on the activity of all types of extracts on the NCI-H460 cell line was weak. Considering the GI_50_ of the ellipticine was 1.03 ± 0.09 μg/ml, the overall activity of SGE and SGI is modest. Moreover, none of the extracts were able to inhibit the PLP2 cell line (up to the maximum assayed concentration: 400 μg/ml extract), which constitutes a good indicator of the lack of toxicity of all the *S. glutinosa* extracts in non-tumor cell lines.

For the determination of anti-inflammatory activity, only the SGE were able to inhibit the production of nitrogen oxide species (NOS). However, the determined IC_50_ values (304.93 ± 11.19 μg/ml for SG1 and 263.07 ± 11.62 μg/ml for SG2) were much higher than the IC_50_ of dexamethasone, which was determined to be 16.0 ± 1.0 μg/ml, which shows a weaker activity in comparison to a compound that is used therapeutically for inflammation.

### 3.6 *In vivo* anti-inflammatory effect

Because polyphenols may act as antioxidants or as prooxidants, some plant extracts have antioxidant properties both *in vitro* and *in vivo*, and for other extracts the *in vitro* antioxidant activity does not apply *in vivo* (Veskoukis et al., 2012)*.* Therefore, after demonstrating the *in vitro* antioxidant capacity using the DPPH, ABTS, and FRAP assays, the *in vivo* antioxidant activity was evaluated through the assessment of several oxidative stress biomarkers, as shown in Table 4.

**TABLE 4:** Determined parameters used in the evaluation of the *in vivo* anti-inflammatory effects of *Salvia glutinosa* extracts.

Groups	TOS µM H_2_O_2_ equiv./L		TAC mM trolox equiv./L		OSI		NOx µM/L		MDA nM/L		SH µM/L	
CONTROL	4.22	±0.57	1.089	±0.003	3.87	±0.52	44.34	±9.25	4.24	±0.42	344.33	±31.85
INFLAM	6.04	±1.32##	1.087	±0.001	5.56	±1.22##	62.09	±5.50##	5.40	±0.25##	203.67	±30.49##
DICLO	3.84	±0.40**	1.091	±0.010	3.52	±0.34**	51.97	±4.41**	4.89	±0.23**	292.92	±45.16**
INFLAMS100	5.05	±0.46**	1.087	±0.001	4.65	±0.43**	46.07	±4.82***	3.33	±0.86***	248.33	±34.08
INFLAMS50	4.95	±0.42**	1.089	±0.002	4.09	±0.38**	55.38	±6.01**	3.31	±0.52***	224.67	±25.61
INFLAMS25	5.08	±0.70**	1.088	±0.002	4.67	±0.64**	54.62	±2.64	2.73	±0.43***	228.33	±41.68
S100	4.29	±0.36	1.086	±0.001	3.87	±0.33	44.39	±5.01	3.21	±0.60##	339.00	±41.93
S50	4.99	±1.38	1.089	±0.002	3.70	±1.41	50.91	±5.30	3.26	±0.59##	330.33	±56.49
S25	4.35	±1.44	1.088	±0.001	3.84	±1.32	50.05	±3.61	3.89	±0.45#	349.00	±34.43

Values are expressed as mean ± SD (*n* = 5); TOS, total oxidative status; TAC, total antioxidant capacity; OSI, oxidative stress Index; NOx, total nitrites and nitrates; MDA, malondialdehyde; SH, total thiols; CONTROL, negative control; INFLAM -inflammation; DICLO, inflammation treated with diclofenac; INFLAMS100—inflammation treated with S. glutinosa extract 100% (200 mg d. w./ml); INFLAMS50—inflammation treated with S. glutinosa extract 50% (100 mg d. w./ml); INFLAMS25—inflammation treated with S. glutinosa extract 25% (50 mg d. w./ml); S100—treated with S. glutinosa extract 100% (200 mg d.w./ml); S50—treated with S. glutinosa extract 50% (100 mg d. w./ml); S25—treated with S. glutinosa extract 25% (50 mg d. w./ml); #*p* ˂ 0.05, ##*p* ˂ 0.01 versus CONTROL; **p ˂ 0.01, ***p ˂ 0.001 versus INFLAM, group.

Inflammation increased significantly oxidative stress by increasing TOS, and OSI (*p* < 0.01). These changes were associated with an elevation of NO (*p* < 0.01) and MDA (*p* < 0.001). At the same time, INFLAM decreased the SH levels (*p* < 0.001) without an important change of TAC (*p* > 0.05). After the induction of inflammation, the administration of *S. glutinosa* extracts reduced the oxidative stress by decreasing TOS and OSI (*p* < 0.01), without significant differences between the three dilutions. When compared to the diclofenac reference, *S. glutinosa* extract showed a lower impact on TOS and OSI (*p* < 0.001). When testing the extract in the group without inflammation, the effect on TOS and OSI was inconsequential (*p* > 0.05).

At the cellular level, ROS are involved in the peroxidation of phospholipids and fatty acids from the cell membrane, which leads to an alteration of membrane fluidity, protein structure, and cell signaling. MDA, a parameter used as a measure for lipoperoxidation, increased in INFLAM (*p* < 0.001). Moreover, the administration of *S. glutinosa* extract after inflammation induction caused an important reduction of MDA (*p* < 0.001), yet with no significant differences between the three doses. Interestingly, administrating *S. glutinosa* extract without inflammation caused a reduction of MDA as well (*p* < 0.001) when compared to the CONTROL group. Furthermore, *S. glutinosa* extract administration with or without inflammation was able to decrease more MDA than diclofenac did (*p* < 0.01). SH levels were not influenced by *S. glutinosa* extract administration, with or without inflammation (*p* > 0.05).

The endogenous nitric oxide (NO) is synthesized by a series of enzymes, one of the most important being the inducible nitric oxide synthases (iNOS). During inflammation and at nontoxic concentrations, NO acts as an antioxidant that protects cells against lipid peroxidation (Balea et al., 2018). However, there is also a toxicity hypothesis, which states that if NO synthesis is excessive, it begins reacting with reactive oxygen species (ROS), producing the peroxynitrite ion (ONOO−). This oxyanion hence acts a strong oxidant species, being able to induce mitochondrial respiratory inhibition and ATP depletion, oxidative, nitrosative, and nitration stress (Balea et al., 2018). NO reduction can be considered as an antioxidant as well as an anti-inflammatory mechanism. Nitric oxide synthesis was evaluated by measuring NOx. In the INFLAM group there was a significant increase (*p* < 0.01), and *S. glutinosa* extract administration after inflammation induction caused a reduction of NOx in a dose dependent way, INFMAS100 having the best inhibitory effect (*p* < 0.001), followed by the INFLAMS50 and INFLAMS25 (*p* < 0.05). Using the *S. glutinosa* extract alone had no relevant effect on NOx (*p* > 0.05). Administration of diclofenac significantly lowered NOx (*p* < 0.01), but the effect was weaker compared to INFMAS100 (*p* < 0.01), but comparable to INFMAS50 and INFMAS25.

Considering all the results, we can reach to the conclusion that in turpentine-induced inflammation, administrating *S. glutinosa* extract displays antioxidant and anti-inflammatory effects. Judging from the determined parameters, the extract could reduce the total oxidants levels, NO and MDA, but it showed no important effect on the antioxidants. Furthermore, prophylactic administration of the same extract when there was no inflammation, caused a reduction of the MDA levels as well.

Moreover, recent studies shined a new light over the importance of natural compounds and their effect on inflammation. Specifically, it has been proven that luteolin and its *O*-glucosides provide anti-inflammatory activity *in vitro* and *in vivo* (Aziz et al., 2018). Thus, there might be a correlation between the existing luteolin acetyl-glucoside in the plant material and the anti-inflammatory effect that has been observed in animals.

ROS are known for being one of the main factors of oxidative stress, thus being involved in developing cardiovascular diseases (stroke and heart attack), neurodegenerative diseases, tumors and diabetes, through chronic inflammation (Balea et al., 2018). The results suggest that the extracts of *S. glutinosa* could be used for prevention and treatment of these pathological conditions.

## 4 Conclusion

Numerous species in the genus *Salvia* have been recently researched to certify their health-related benefits. Taking into consideration the necessity to improve the knowledge regarding lesser known Lamiaceae species, we have focused this study on the comparative phytochemical and bioactivity analysis of extracts obtained from *S. glutinosa* samples collected from two different locations.

*S. glutinosa* aerial parts HPLC phytochemical analysis showed a polyphenol-rich composition, with important quantities of rosmarinic acid and luteolin acetyl-glucoside in the aqueous and the hydroalcoholic extracts, as well as some types of *O*-hexosides, with differences related to the geographical source. These compounds seem to be responsible for the significant *in vitro* antioxidant and α-glucosidase inhibitory activities and for the modest antiproliferative effect on HepG2 cell line. The powders obtained from the hydroalcoholic extracts showed a higher activity compared to the infusions. The *in vivo* anti-inflammatory and antioxidant effects in a turpentine-induced inflammation rat model were associated with oxidants reduction. Furthermore, prophylactic administration of *S. glutinosa* extract increased serum antioxidants. To our knowledge, this study represents the first *in vivo* research regarding the bioactivity of *S. glutinosa*.

Further research evidence, through established preclinical and clinical studies, would be required to confirm the therapeutic anti-inflammatory and antidiabetic effects of SG and to establish possible prophylactic antioxidant activity.

## Data Availability

The original contributions presented in the study are included in the article/Supplementary Material, further inquiries can be directed to the corresponding author.
