# Supplementary figures and images for: Potential therapeutic applications of infusions and hydroalcoholic extracts of Romanian glutinous sage (Salvia glutinosa L.)
Source: Front Pharmacol. 2022 Aug 19;13:975800. doi: 10.3389/fphar.2022.975800 (PMC9437640; doi:10.3389/fphar.2022.975800)

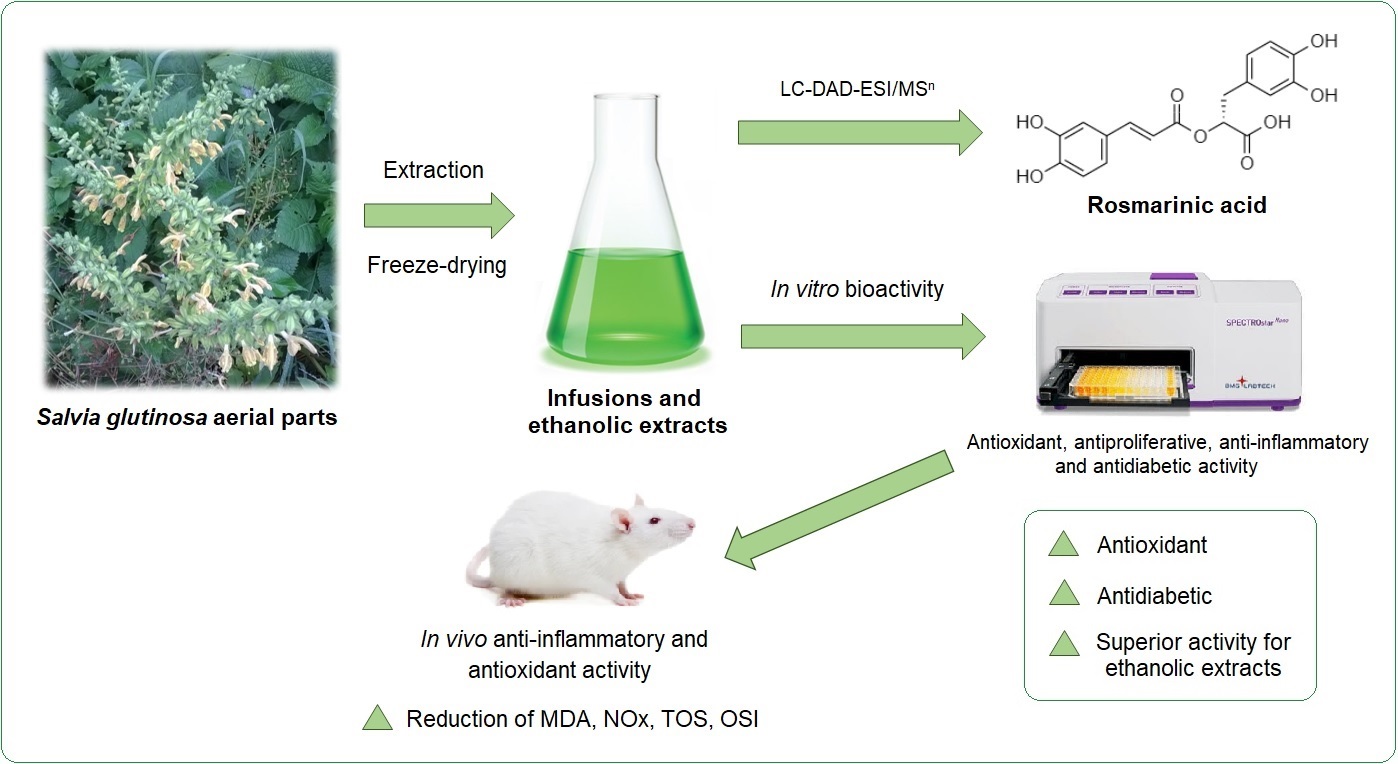

Supplement: Supplementary file 1 [file Image1.JPEG]
