# Supplementary material for: Potential therapeutic applications of infusions and hydroalcoholic extracts of Romanian glutinous sage (Salvia glutinosa L.)
Source: Front Pharmacol. 2022 Aug 19;13:975800. doi: 10.3389/fphar.2022.975800 (PMC9437640; doi:10.3389/fphar.2022.975800)
Supplement: Supplementary file 2 [file DataSheet1.docx]

**Supplementary material**

| **SG1** | **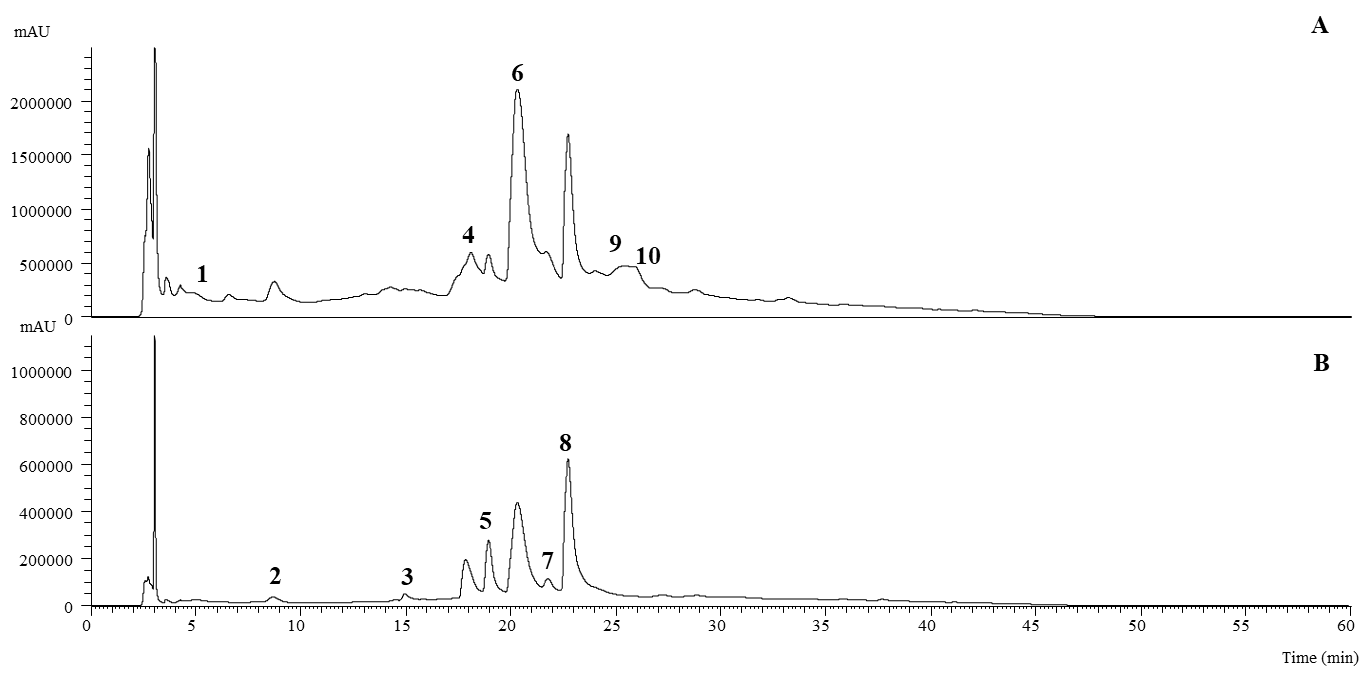** |
| --- | --- |
| **SG2** | **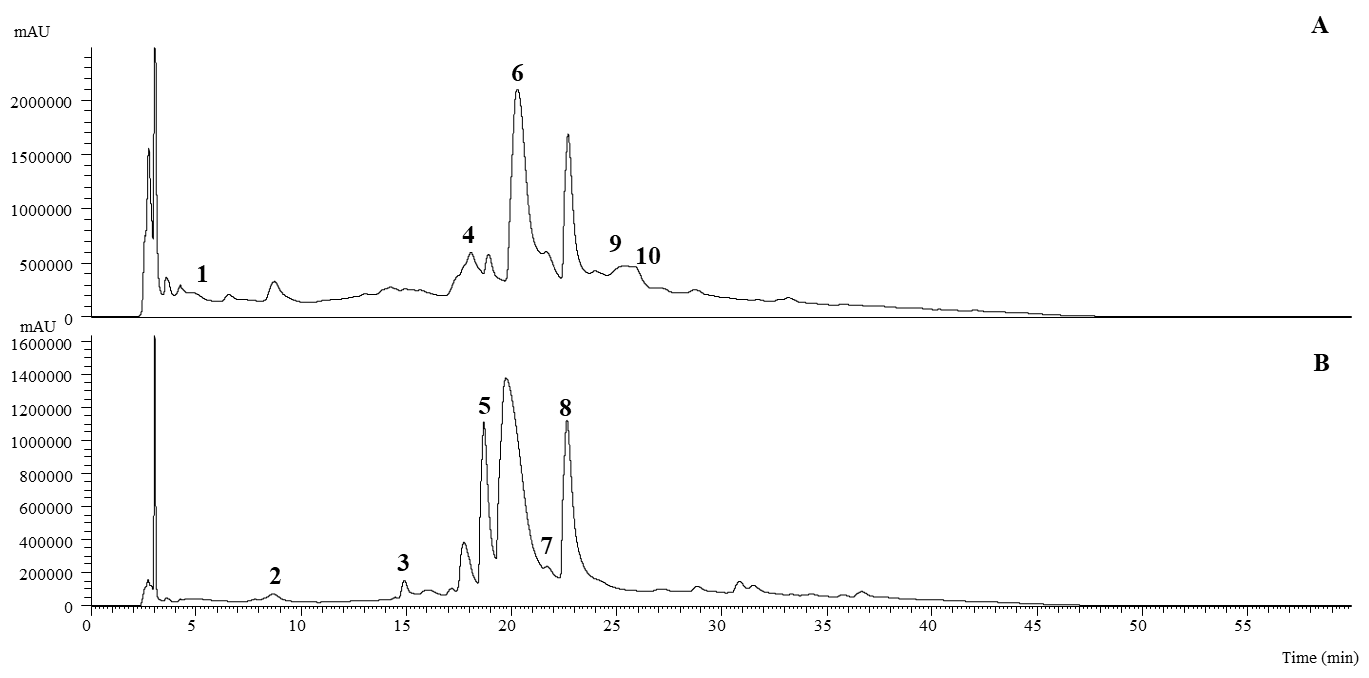** |
| **SG3** | **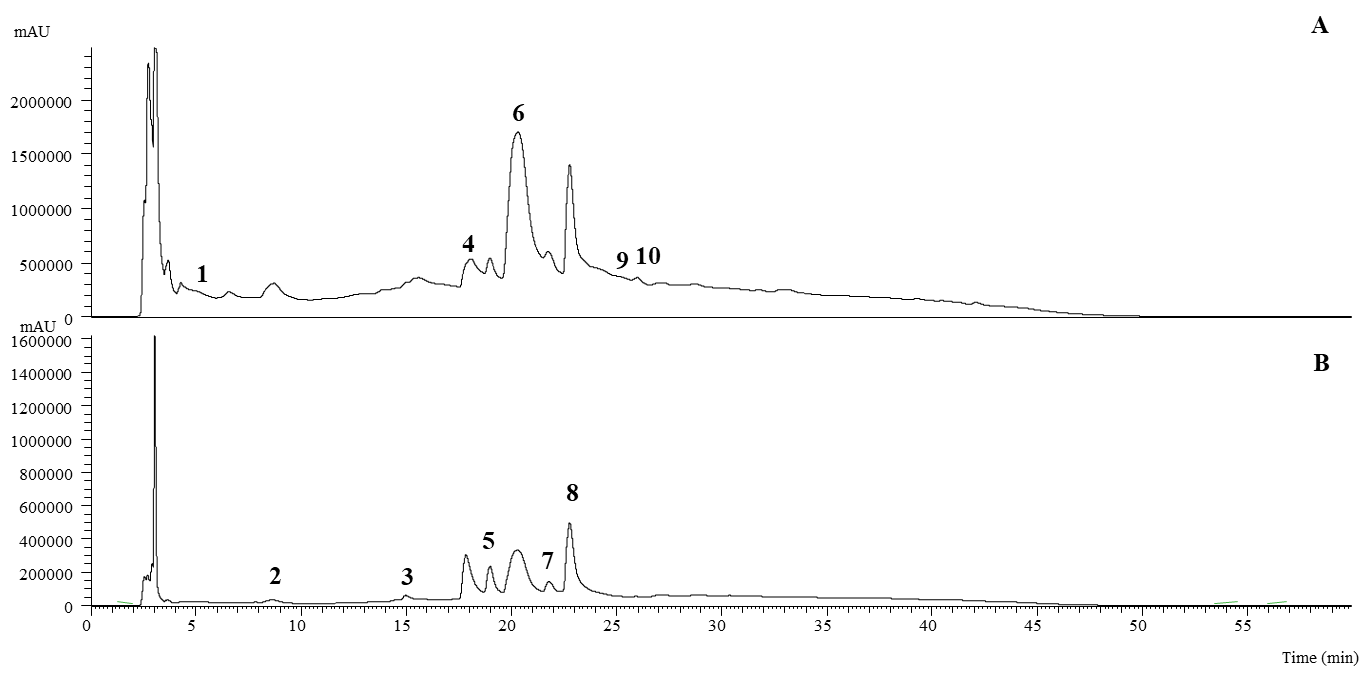** |
| **SG4** | **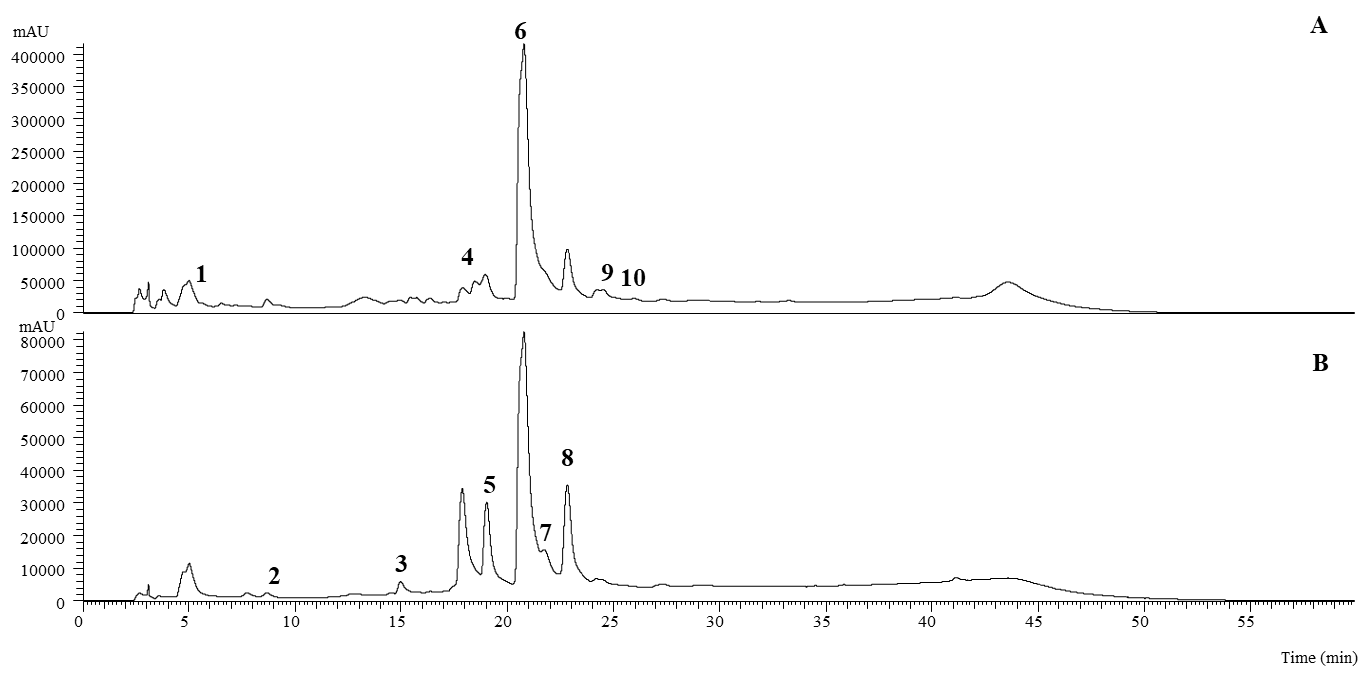** |

**SM1.** Exemplificative phenolic profiles of SG1, SG2, SG3, SG4 samples recorded at 280 nm (A) and 370 nm (B).

**SM2**. UV spectra, pseudomolecular ion and mass fragmentation pattern of the four standard compounds used for quantification, apigenin-6-C-glucoside, caffeic acid, rosmarinic acid, and quercetin-3-*O*-glucoside.

| **Standard compound used**  **for quantification** | **λ_máx_**  **(nm)** | **UV spectrum**  **Representation** | **Deprotonated ion**  **[M-H]^-^** | **MS^2^ fragmentation pattern**  **(*m/z*)** |
| --- | --- | --- | --- | --- |
| **Apigenin-6-*C*-glucoside** | 336 | 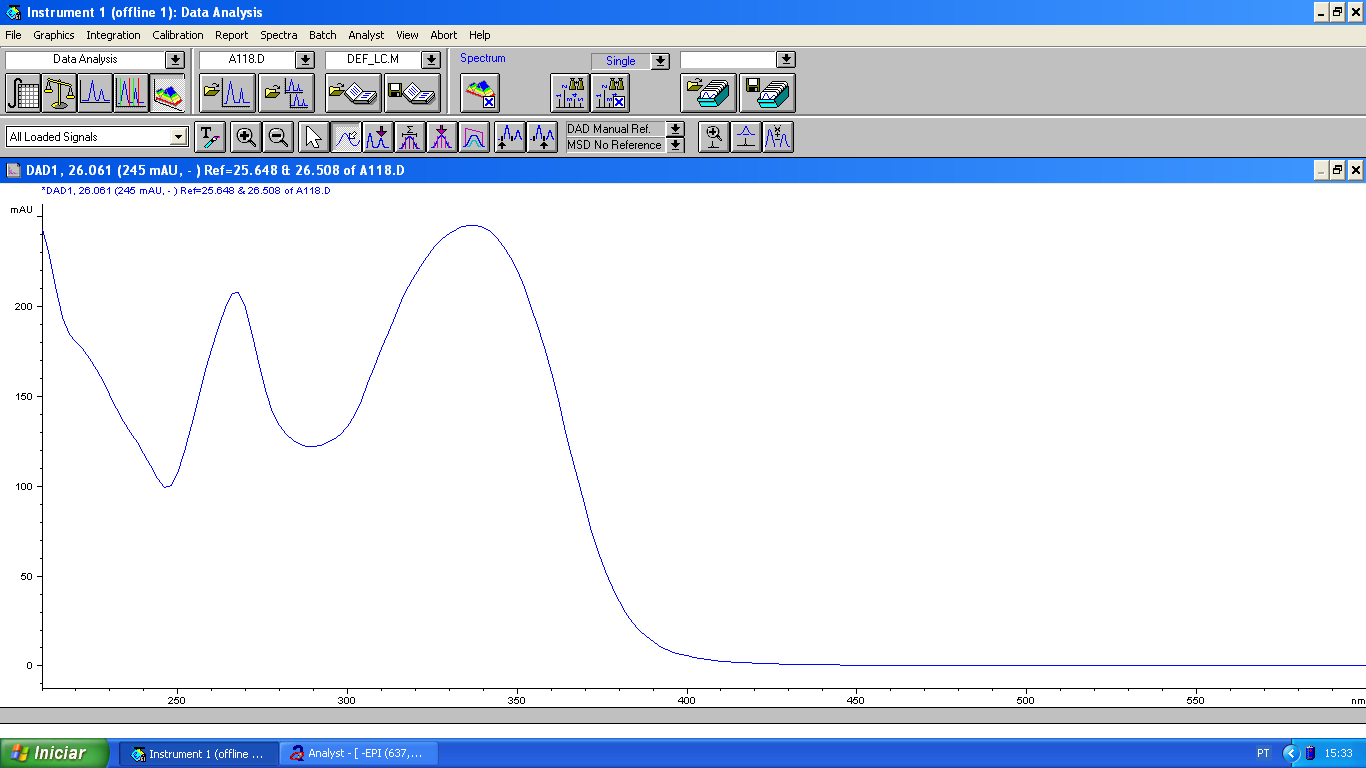 | 431 | 413(13),341(48),311(100) |
| **Caffeic acid** | 328 | 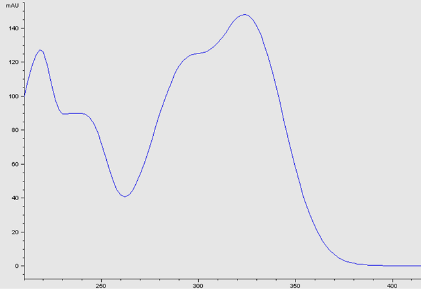 | 179 | 135(100) |
| **Rosmarinic acid** | 330 | 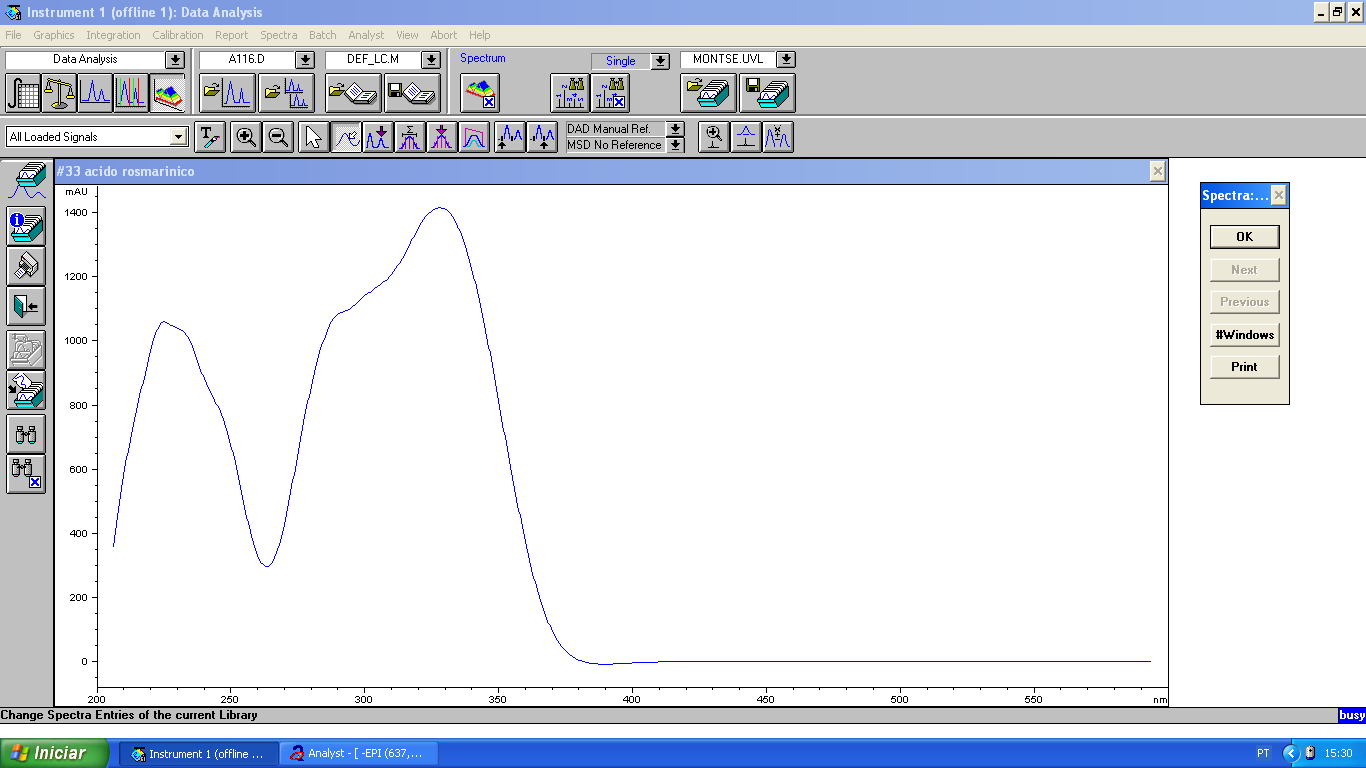 | 359 | 197(98), 179(94), 161(100), 135(58) |
| **Quercetin 3-*O*-glucoside** | 354 | 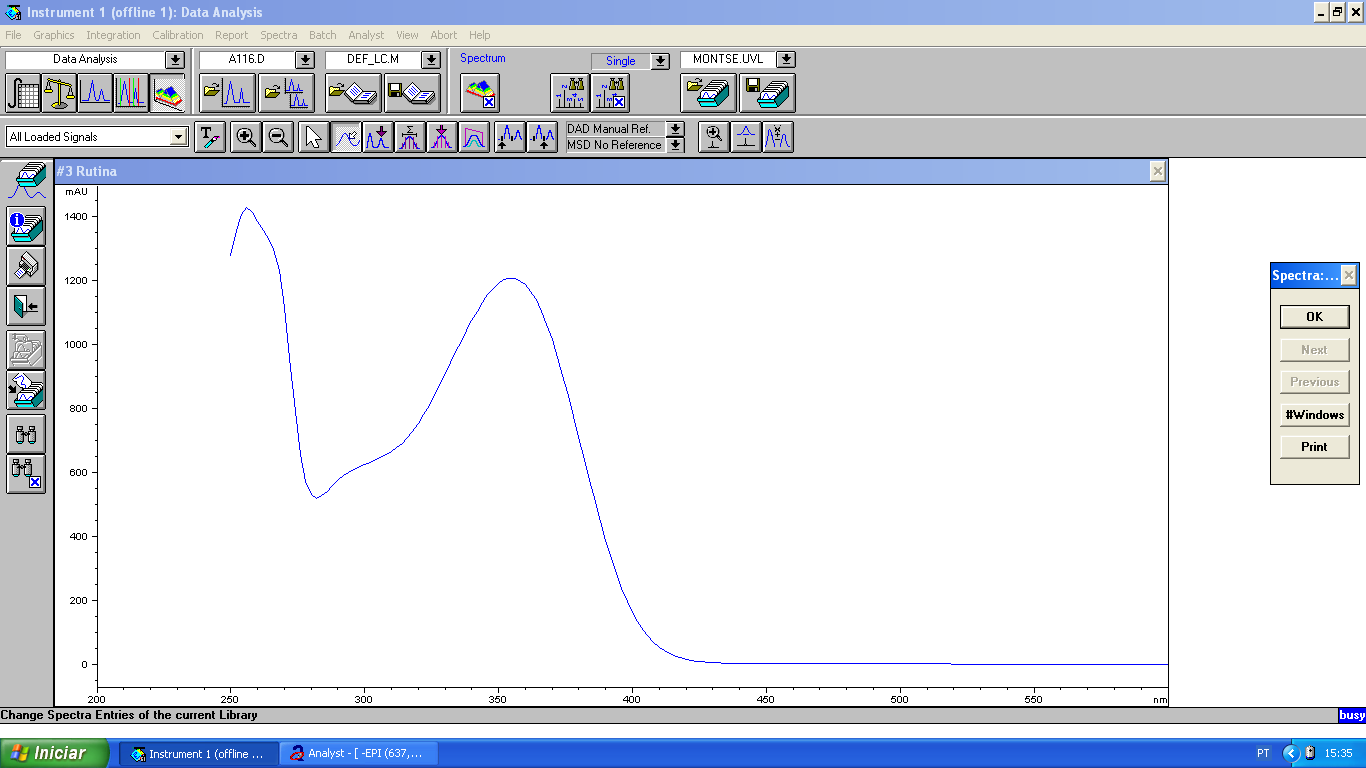 | 463 | 301(100) |
